# Virus RNA Load in Patients with Tick-Borne Encephalitis, Slovenia

**DOI:** 10.3201/eid2407.180059

**Published:** 2018-07

**Authors:** Ana Saksida, Nina Jakopin, Mateja Jelovšek, Nataša Knap, Luka Fajs, Lara Lusa, Stanka Lotrič-Furlan, Petra Bogovič, Maja Arnež, Franc Strle, Tatjana Avšič-Županc

**Affiliations:** University of Ljubljana, Ljubljana Faculty of Medicine, Slovenia (A. Saksida, N. Jakopin, M. Jelovšek, N. Knap, L. Fajs, L. Lusa, T. Avšič-Županc);; University Medical Center Ljubljana, Ljubljana (S. Lotrič-Furlan, P. Bogovič, M. Arnež, F. Strle)

**Keywords:** tickborne encephalitis virus, (TBEV), viruses, arboviruses, flavivirus, arbovirus infections, tickborne diseases, tick-borne encephalitis, ticks, meningitis/encephalitis, virus RNA, virus load, vector-borne infections, Slovenia

## Abstract

We determined levels of tick-borne encephalitis (TBE) virus (TBEV) RNA in serum samples obtained from 80 patients during the initial phase of TBE in Slovenia. For most samples, levels were within the range of 3–6 log_10_ copies RNA/mL. Levels were higher in female patients than in male patients, but we found no association between virus load and several laboratory and clinical parameters, including severity of TBE. However, a weak humoral immune response was associated with a more severe disease course, suggesting that inefficient clearance of virus results in a more serious illness. To determine whether a certain genetic lineage of TBEV had a higher virulence potential, we obtained 56 partial envelope protein gene sequences by directly sequencing reverse transcription PCR products from clinical samples of patients. This method provided a large set of patient-derived TBEV sequences. We observed no association between phylogenetic clades and virus load or disease severity.

Tick-borne encephalitis (TBE) is one of the major virus infections of the human central nervous system (CNS) in Europe and Asia. This disease is caused by TBE virus (TBEV) (family *Flaviviridae*, genus *Flavivirus*). Three main subtypes of this virus have been recognized (European, Siberian, and Far Eastern), and their geographic distribution closely resembles the distribution of their tick vectors, namely *Ixodes ricinus* ticks for the European subtype and *I. persulcatus* ticks for the Siberian and Far Eastern subtypes (*1*,*2*).

Approximately 10,000–15,000 TBE cases are reported annually; 3,000 of them are in Europe. However, because reporting of TBE is not established in all disease-endemic countries, the real numbers are most likely higher (*2*,*3*). Humans acquire TBEV infection mainly through tick bites and only rarely (≈1%) by consuming unpasteurized milk or milk products from infected livestock, particularly goats (*4*–*6*). Thus, most TBE cases occur in the warm months of the year (April–November), which corresponds with the main period of tick activity (*7*,*8*).

Most (70%–98%) TBEV infections are believed to be asymptomatic (*9*,*10*). In ≈75% of patients with TBE caused by the European subtype, the disease has a typical biphasic course. The first phase, which follows an incubation period with a median of 8 days (range 2–28 days) after a tick bite, and which correlates with viremia, is characterized by nonspecific symptoms, such as fever, fatigue, general malaise, headache, and body pain, which are often associated with leukopenia or thrombocytopenia. The initial phase lasts for 2–7 days and is followed by an improvement or even an asymptomatic interval of ≈1 week (range 1–21 days). The second phase manifests as meningitis (≈50% of adult patients), meningoencephalitis (≈40%), or meningoencephalomyelitis (≈10%) (*11*–*13*). The severity of TBE increases with the age of patients (*14*,*15*). Unfavorable outcomes, including long-term sequelae, are more often seen in patients with severe acute illness (*16*,*17*) and other clinical and laboratory findings (*12*,*16*,*18*). However, the exact mechanisms leading to more severe disease and unfavorable outcome in an individual patient are not known.

After a tick bite, TBEV replication occurs locally in dendritic skin cells. From there, the virus reaches other organs, especially the spleen, liver, and bone marrow. It is believed that production of high levels of virus in the affected organs, resulting in viremia, is a prerequisite for the virus to cross the blood–brain barrier because the capillary endothelium is not easily infected. However, the exact mechanism by which TBEV accesses the brain is not known (*13*,*19*,*20*). Furthermore, some authors reported a correlation between a low concentration of neutralizing antibodies and more severe disease, suggesting that a delayed formation of neutralizing antibodies could be associated with high viremia (*16*,*21*). However, no information is available on the level of viremia in patients with TBE and its effect on disease severity. The main purpose of this study was to determine levels of TBEV RNA in clinical samples of patients with TBE and correlate these levels with several laboratory and clinical parameters, including severity of the disease.

## Patients and Methods

### Patients and Samples

Patients eligible for study were those given a diagnosis of TBE at the Department of Infectious Diseases, University Medical Center Ljubljana (Ljubljana, Slovenia), during 2003–2013 who were seen at the initial and second (meningoencephalitic) phases of TBE and in whom TBEV was identified by PCR in serum specimens obtained during the initial phase of the disease. Initial-phase serum samples were obtained either during a prospective study on the etiology of febrile illness after a tick bite or represented remnants of the samples collected as a part of routine diagnostic testing of a patient with a febrile illness in whom TBE later developed. In addition, available cerebrospinal fluid (CSF) samples obtained during the meningoencephalitic phase of the illness from the same patients were also included in the study. The specimens were stored at −80°C until further processing.

### Definitions

The initial phase of TBE was classified as a febrile illness that, after a clinical improvement lasting <21 days, was followed by neurologic involvement. TBE was defined as clinical signs or symptoms of meningitis or meningoencephalitis, increased CSF leukocyte counts (>5 × 10^6^ cells/L), and serum TBEV IgM and IgG or TBEV IgG seroconversion in paired serum samples. TBE was categorized as mild (only signs or symptoms of meningeal involvement), moderate (monofocal neurologic signs or mild-to-moderate signs/symptoms of CNS dysfunction), or severe (multifocal neurologic signs or signs/symptoms of severe dysfunction of the CNS) (*11*).

In addition to this simple clinical classification, a quantitative evaluation of the severity of the disease was performed by using a standardized questionnaire as reported (*22*). Points (1–9) were assigned for the presence, intensity, and duration of headache, fever, vomiting, and meningeal signs; the presence of tremor, pareses, urine retention, and cognitive function disturbances; the presence and intensity of conscious disturbances; and the need for and duration of treatment for increased intracranial pressure. The absence of a particular symptom/sign scored. A score <9 corresponded to clinically mild disease, 9–22 to moderate disease, and >22 to severe disease (*22*).

### Ethics Considerations

The study was conducted according to the principles of the Declaration of Helsinki, the Oviedo Convention on Human Rights and Biomedicine, and the Slovene Code of Medical Deontology. The study was approved by the National Medical Ethics Committee of Slovenia (no. 152/06/13, no. 178/02/13, and no. 37/12/13). Patients whose specimens were obtained in the study on the etiology of febrile illness after a tick bite signed an informed consent form that included the use of collected specimens for further studies. The Ethics Committee waived the need for written informed consent for patients for whom remnants of routinely collected serum specimens were used.

### TBEV Antibody Levels

We determined the presence and concentration of TBEV antibodies in serum samples by using the Enzygnost Anti-TBE/FSME Virus (IgM, IgG) test (Siemens AG, Munich, Germany) according to the manufacturer’s instructions. Specificities of the test were 99.5% for IgG and 99.9% for IgM, and sensitivities were 96.8% for IgG and 98.8% for IgM.

### TBEV RNA Load

We extracted total RNA from serum and CSF samples by using the QIAamp Viral RNA Mini Kit (QIAGEN, Hilden, Germany) according to the manufacturer’s instructions. For a quantitative reverse transcription PCR (RT-PCR), we used the TaqMan Fast Virus 1-Step Master Mix (Applied Biosystems, Carlsbad, CA, USA). This RT-PCR was performed as reported (*23*). For analysis purposes, we converted virus loads to log_10_ values.

### Sequencing and Phylogenetic Analysis

We obtained sequences by direct sequencing of RT-PCR products from serum samples of patients with TBE. A partial TBEV envelope (E) protein gene was amplified and sequenced by using primer pair TBE ENV 3F (5′-TGA GGG GAA GCC TTC AAT-3′) and TBE ENV 3R (5′-TCA TGT TCA GGC CCA ACC A-3′), and sequence analysis was performed as reported (*24*).

### Statistical Analysis

Numerical data were summarized as means and SDs or medians and interquartile ranges (IQRs) and categorical variables as frequencies and percentages. We calculated 95% CIs for means or percentages of some variables. We assessed the association between variables and TBEV RNA by using univariate linear regression; log_10_-transformed TBEV RNA counts were used as the outcome variable. We used a similar approach to assess the association between phylogenetic clades and severity of disease.

We displayed observed associations with outcome variables graphically by using box and whisker plots for categorical variables and scatter plots for numerical variables. We added a loess regression (locally weighted scatterplot smoothing) line (*25*) with 95% CIs fitted by using the geom_smooth function in the ggplot2 R software (*26*). We conducted analyses by using R statistical language (*27*).

## Results

### Patients and Samples

We obtained basic demographic data, clinical characteristics, and laboratory findings for 80 patients with established TBEV RNA in serum during the initial phase of TBE (Table). Second-phase CSF samples were available for 48 of these 80 patients.

**Table:** Characteristics of 80 patients with TBEV RNA in serum obtained during initial phase of TBE, Slovenia*

Characteristic	Value	95% CI†
Sex		
F	43 (53.8)	42.2–64.9
M	37 (46.2)	35.0–57.8
Median age, y (IQR)	48.5 (31–60.8)	42.0–50.5
F	52 (30–63)	41.1–53.2
M	47 (33–59)	39.1– 51.4
History of tick bite	68 (85.0)	75.3–92.0
Initial (first) phase of TBE		
Leukopenia‡	71 (88.8)	79.7–94.7
Thrombocytopenia§	52 (65.0)	53.5–75.3
Duration of first phase (days), median (IQR)	6 (5–8)	5.9–6.7
Asymptomatic interval		
Duration, d, median (IQR)	10 (7–13)	9.5–11.7
Second (meningoencephalitic) phase of TBE		
CSF findings		
Cell count × 10^6^/L, median (IQR)	57 (25–104.8)	64.6–122.6
Protein concentration, mg/L, median (IQR)	0.54 (0.40–0.74)	0.54–0.68
Serum antibodies to TBEV¶		
IgM	80 (100.0)	95.5–100.0
IgG	71 (88.7)	79.7–94.7
Severity of acute illness		
Quantitative assessment		
Mild	28 (35.0)	24.7–46.5
Moderate	45 (56.3)	44.7–67.3
Severe	7 (8.7)	3.6–17.2
Clinical classification		
Mild	29 (36.2)	25.8–47.8
Moderate	45 (56.3)	44.7–67.3
Severe	6 (7.5)	2.8–15.6

*Values are no. (%) patients unless otherwise noted. CSF, cerebrospinal fluid; IQR, interquartile range; TBE, tick-borne encephalitis; TBEV, TBE virus. †For the population percentage or the population mean. ‡Blood leukocyte count <4 × 10^9^/L. §Blood platelet count <140 × 10^9^/L. ¶Findings obtained during second (meningoencephalitic) phase of illness at initial examination; all IgG-negative patients later seroconverted.

### TBEV RNA Load

TBEV RNA was detected in serum of 80 patients with febrile illness in whom neurologic involvement later developed and who fulfilled criteria for TBE. CSF samples obtained at the meningoencephalitic phase of illness from 48 of these 80 patients all showed negative results for TBEV RNA. On the day of virus load detection, only 3 patients were positive for TBE IgM in the serum, and all other patients were negative for TBEV IgM and IgG.

**Figure 1:**
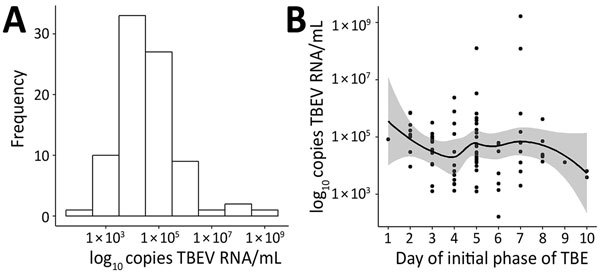
Distribution of virus RNA load in patients with TBE, Slovenia (A), and by day of initial phase of TBE (B). Solid line indicates a loess regression line, and shaded area indicates 95% CIs. TBE, tick-borne encephalitis; TBEV, TBE virus.

The mean (SD) of the logarithmic transformation of TBEV RNA levels in serum was 4.65 (1.13) log_10_ copies RNA/mL. For most (95%) patients, this level showed a range of 3–6 log_10_ copies RNA/mL (Figure 1, panel A). RNA-positive serum results were obtained as early as the first day and as late as the tenth day (median fifth day) day of the initial phase of TBE. For 1 patient, we detected virus RNA in the serum sample on day 14 of disease, which corresponded clinically to the seventh day of the asymptomatic interval. We did not find any substantial differences in numbers of RNA copies within this time frame (Figure 1, panel B).

**Figure 2:**
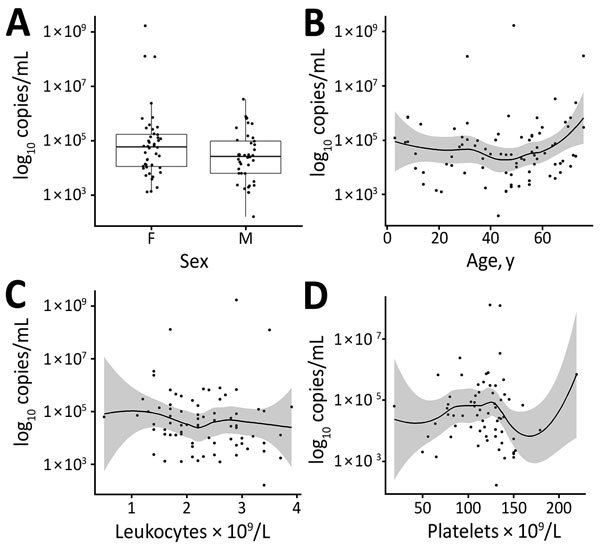
Distribution of virus RNA load in patients with tick-borne encephalitis, Slovenia, by patient sex (A), age (B), leukocyte count (C), and platelet count determined on the same day as RNA load (D). Boxes in panel A indicate interquartile ranges and 25th and 75th percentiles, horizontal lines within boxes indicate medians, and errors bars indicate 1.5× interquartile ranges. Solid lines in panels B–D indicate loess regression lines, and shaded areas indicate 95% CIs.

**Figure 3:**
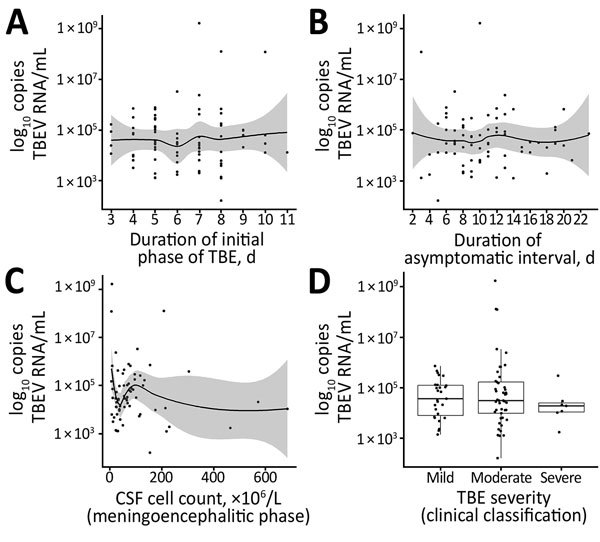
Distribution of virus RNA load in patients with TBE, Slovenia, by duration of initial phase of TBE (A), duration of asymptomatic interval (B), CSF cell count determined in the meningoencephalitic phase (C), and severity of TBE according to clinical classification (D). Solid lines in panels A–C indicate loess regression lines, and shaded areas indicate 95% CIs. Boxes in panel D indicate interquartile ranges and 25th and 75th percentiles, horizontal lines within boxes indicate medians, and error bars indicate 1.5× interquartile ranges. CSF, cerebrospinal fluid; TBE, tick-borne encephalitis; TBEV, TBE virus.

TBEV RNA levels were higher in female patients than in male patients (mean [SD] 4.86 [1.25] vs. 4.4 [0.93] log_10_ copies RNA/mL; p = 0.064). The log-transformed number of detected RNA copies did not appear to be associated with ages of patients, leukocyte and platelet counts determined on the same day as RNA load (Figure 2, panels A–D), duration of the initial phase of TBE, duration of the asymptomatic interval between the initial phase and second phase of TBE, CSF cell count determined in the meningoencephalitic phase of illness, severity of TBE according to quantitative assessment, and simple clinical classification (mean [SD] virus load values in patients with clinically mild, moderate, and severe disease were 4.54 [0.75], 4.68 [1.27], and 4.74 [1.71] log_10_ copies RNA/mL, respectively; p = 0.856) (Figure 3, panels A–D). Associations between these variables and log-transformed TBEV RNA in the serum were not statistically significant, and they did not appear to have any potential clinical role.

**Figure 4:**
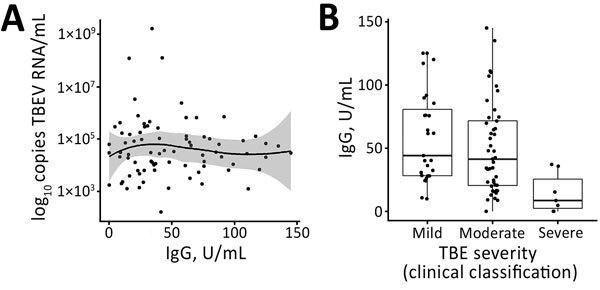
A) Distribution of virus RNA load in patients with TBE, Slovenia, by levels of TBEV IgG. B) Levels of TBEV IgG according to disease severity (clinical classification). Solid line in panel A indicates loess regression line, and shaded area indicates 95% CIs. Boxes in panel B indicate interquartile ranges and 25th and 75th percentiles, horizontal lines within boxes indicate medians, and errors bars indicate 1.5× interquartile ranges. TBE, tick-borne encephalitis virus; TBEV, TBE virus.

We observed no differences in distribution of detected virus RNA levels when compared with concentrations of specific TBE IgG in initial follow-up serum samples of patients obtained during the second phase of the disease (Figure 4, panel A). However, we observed a significant association between TBEV antibody titers and disease severity (p = 0.005); on average, lower IgG levels were observed for patients with more severe illness (Figure 4, panel B).

### Phylogenetic Analysis

We obtained 56 partial E protein gene sequences by direct sequencing of RT-PCR products from serum samples of patients with TBE and used a 1,272-bp segment of the E protein gene for phylogenetic analysis. Analysis showed that in Slovenia, sequences grouped into 6 clades (S1–S6). Nucleotide sequence identity was 95.8%–100% (divergence 0%–4.2%) and amino acid identity was 98.1%–100% (divergence 0%–1.9%). To assess a potential association of sequence divergence with geographic locations, we plotted permanent residencies of patients on a map of Slovenia according to their respective phylogenetic clustering on the basis of E protein gene sequence analysis. Results showed a high level of regional clustering (Technical Appendix Figure).

**Figure 5:**
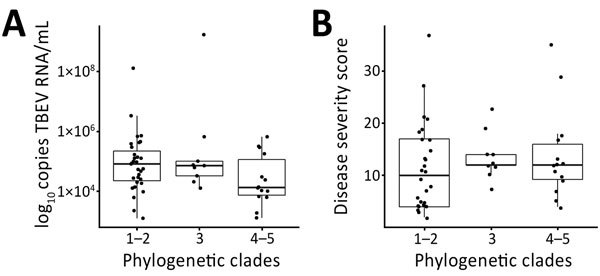
Distribution of virus RNA load in patients with tick-borne encephalitis, Slovenia, by A) virus phylogenetic clades and B) disease severity scores according to Bogovic et al. (*22*). Boxes indicate interquartile ranges and 25th and 75th percentiles, horizontal lines within boxes indicate medians, and errors bars indicate 1.5x interquartile ranges. TBEV, tick-borne encephalitis virus.

To determine whether a certain genetic lineage of TBEV had a higher virulence potential, we compared phylogenetic clades with levels of TBEV RNA and disease severity. Because there were too many groups to perform a meaningful statistical analysis, we combined some clades on the basis of their sequence identities (Figure 5). Although levels of TBEV RNA were somewhat lower in patients infected with viruses belonging to S4 and S5 phylogenetic clades, differences were not significant (p = 0.116). Also, we observed no association between phylogenetic clades and disease severity (Figure 5).

## Discussion

Although TBE is one of the major and serious neuroinfections in Europe and Asia, some crucial steps in the development of the disease remain poorly understood. It has been postulated that high virus replication in the primarily affected organs, which maintains viremia in the first phase of the disease, is a prerequisite for the virus to cross the blood–brain barrier because viruses with a low capacity to generate viremia in peripheral tissue can be classified as having low neuroinvasiveness, regardless of their intrinsic neurovirulence potential (*13*,*19*,*20*). However, levels of viremia have so far been reported only in a few (individual) TBEV-infected patients (*23*,*28*).

In our study, we determined TBEV RNA loads in clinical samples and their association with laboratory and clinical parameters in a large group of patients with TBE. TBEV RNA levels were measured and detected in 80 first-phase serum samples obtained from 80 patients in whom neurologic involvement later developed and who were hospitalized for TBE during 2003–2013. TBEV RNA was detected in the time frame of 1–14 days from the beginning of the initial phase of illness. At the time of TBE RNA measurement, 79 patients were febrile (i.e., they were in the initial phase of TBE clinically). For 1 patient, TBEV RNA was detected 7 days after defervescence and 8 days before recurrence of fever; thus, this patient was clinically interpreted to be in the asymptomatic interval. For 95% of patients, levels of TBEV RNA were 3–6 log_10_ copies/mL and showed a mean (SD) value of 4.65 (1.13) log_10_ copies RNA/mL. In comparison, levels of viremia in blood or plasma samples of persons infected with West Nile virus (WNV) were somewhat lower (mean values 3–4 log_10_ copies RNA/mL), but higher virus loads have been reported in urine samples (*29*–*32*). However, studies on WNV virus load included patients with symptomatic (West Nile fever and West Nile neurologic disease), as well as patients with asymptomatic infections; all of our patients were symptomatic and had neurologic manifestations. Data are not available for viremia levels in patients infected with Japanese encephalitis virus, another major neurotropic flavivirus.

Although we determined that TBEV RNA was readily detected in first-phase TBE serum samples, TBEV RNA could not be detected in any of the second-phase CSF samples. This finding confirms previous findings that PCR examination of serum is a valuable approach for a diagnosis of TBEV infection in the first phase of the disease, whereas corresponding testing of CSF obtained in the meningoencephalitic phase of TBE is not a valuable approach (*33*).

We detected higher levels of TBEV RNA in female patients than in male patients. However, we found no association with age of patients. We do not have an obvious explanation for higher viremia in female patients, but female patients did not differ from male patients by age, day of illness when virus load was measured, severity of disease, and several other parameters.

To evaluate the role of TBEV RNA levels in the pathogenesis of disease, we assessed the association between measured TBEV RNA load and several clinical parameters, including duration of the first phase of the disease, duration of the asymptomatic interval, TBE severity, and clinical presentation. However, univariate linear regression showed no association between virus load and any of the observed variables (Figure 3). This finding is consistent with findings from studies of laboratory mice, in which no association was detected between levels of TBEV, as well as WNV viremia and survival rates of animals (*34*,*35*). Studies of humans infected with WNV showed that higher virus loads in blood, plasma, or urine were present in patients with symptomatic infections than in those with asymptomatic infections, but no association was found between WNV burden and disease course (*29*,*31*,*32*,*36*). Because our study included only patients with symptomatic infection (i.e., those with biphasic TBE), we were unable to compare virus loads of the 2 viruses in asymptomatic infections.

Furthermore, our study found no association between virus RNA levels and laboratory parameters measured during the course of the disease, including leukocyte and platelet counts determined on the same day as RNA load and CSF cell counts and TBEV IgG levels determined at the beginning of encephalitic phase of TBE. However, a strong association was observed when TBEV IgG levels were compared with disease severity; the highest concentrations of antibodies were detected in patients with a mild form of the disease and the lowest concentrations were detected in patients with a severe form of the disease. This finding might suggest that although higher levels of TBEV RNA are not directly associated with a more severe disease course, a limited or delayed humoral response results in a more severe illness caused by failure of the host to clear the virus. Thus, such prolonged viremia could result in a more pronounced infection of neuronal cells and subsequently in a more severe clinical presentation. This hypothesis is supported by previous studies of TBE patients (*16*) and experimentally infected laboratory animals (*21*,*35*,*37*), in which low concentrations of TBEV-specific antibodies and TBEV neutralizing antibodies in serum coincided with subsequent appearance of TBE and more severe disease.

Severity of TBE has been reported to vary in different geographic regions because severity is related to the subtype of the virus causing the infection. Thus, disease caused by the European TBEV subtype is considered to be milder than TBE caused by Siberian and Far Eastern subtypes, for which higher case-fatality rates and severe neurologic sequelae rates have been reported (*1*,*38*). However, differences in clinical presentation of TBE have also been reported in areas where only 1 virus subtype was present (*11*,*17*,*39*).

In our study, we obtained 56 partial E protein gene sequences by directly sequencing RT-PCR products from clinical samples of patients to avoid occurrence of mutations that could arise in the process of virus culturing or cloning. Relatively high genetic variability of TBEV from Slovenia was observed, which corroborates the results of Fajs et al. for a smaller number of patients in Slovenia (*24*). Previous studies of genetic diversity of TBEV have also shown that multiple sequence variants are present in relatively small geographic areas (*40*–*43*). In our study, we identified 6 phylogenetic clades by analyses of patient-derived E protein gene sequence analyses. Although the phylogeographic analysis of human samples included locations of residence, which do not necessarily correlate with site of infection, we observed an association between geographic and phylogenetic clustering, which suggested that most patients become infected near their homes, as reported previously (*44*). However, we found no association between phylogenetic clades and levels of TBEV RNA. Somewhat lower levels were detected in patients infected with viruses belonging to S4 and S5 phylogenetic clades, but we observed no major differences.

We also observed no association between phylogenetic clades and disease severity. These results do not demonstrate that differences in clinical presentation of TBE observed in a small geographic area are attributable to different genetic variants of the virus circulating in the area. Although in studies conducted by Belikov et al. (*45*) and Leonova et al. (*46*) in which full-genome sequences of Far Eastern TBEV strains isolated from patients with variable disease severity were analyzed, these authors found that the position of the strain on the phylogenetic tree and presence of specific mutations showed a strong correlation with pathogenicity of TBEV strains and disease severity; mutations found in the E protein gene sequence did not correlate with the degree of pathogenicity of TBEV strains. Also, other studies reported that mutations in genome regions other than E protein gene could be responsible for changes in neuroinvasivenes and neurovirulence (*47*–*49*).Therefore, phylogenetic analysis of more genome sequences is needed for better understanding of potential differences in pathogenicity of virus strains circulating in Slovenia.

In conclusion, for most patients with TBE, levels of TBEV RNA in serum in the first stage of illness are 3–6 log_10_ copies RNA/mL. The findings of our study do not indicate that levels of TBEV RNA in the first stage of TBE are directly associated with clinical presentation or severity of disease, or that levels can be used as a prognosis factor. Nevertheless, a weak humoral immune response seems to be associated with more severe acute illness, which suggests that inefficient clearance of virus results in a more serious infection of the CNS. The pathogenesis of TBE is most likely a complex and multifactorial process driven by properties of TBEV, as well as by the immune responses of the host. Further studies are needed to substantiate the association between the efficiency of virus clearance, prolonged viremia, and clinical presentation, and to better elucidate the immunopathogenesis of infections with TBEV.

Technical Appendix Additional information on virus RNA load in patients with tick-borne encephalitis, Slovenia.
