# Supplementary material for: Virus RNA Load in Patients with Tick-Borne Encephalitis, Slovenia
Source: Emerg Infect Dis. 2018 Jul;24(7):1315–23. doi: 10.3201/eid2407.180059 (PMC6038823; doi:10.3201/eid2407.180059)
Supplement: Technical Appendix — Additional information on virus RNA load in patients with tick-borne encephalitis, Slovenia. [file 18-0059-Techapp-s1.pdf]

Article DOI: https://doi.org/10.3201/eid2407.180059

Virus RNA Load in Patients with Tick-Borne Encephalitis, Slovenia

Technical Appendix

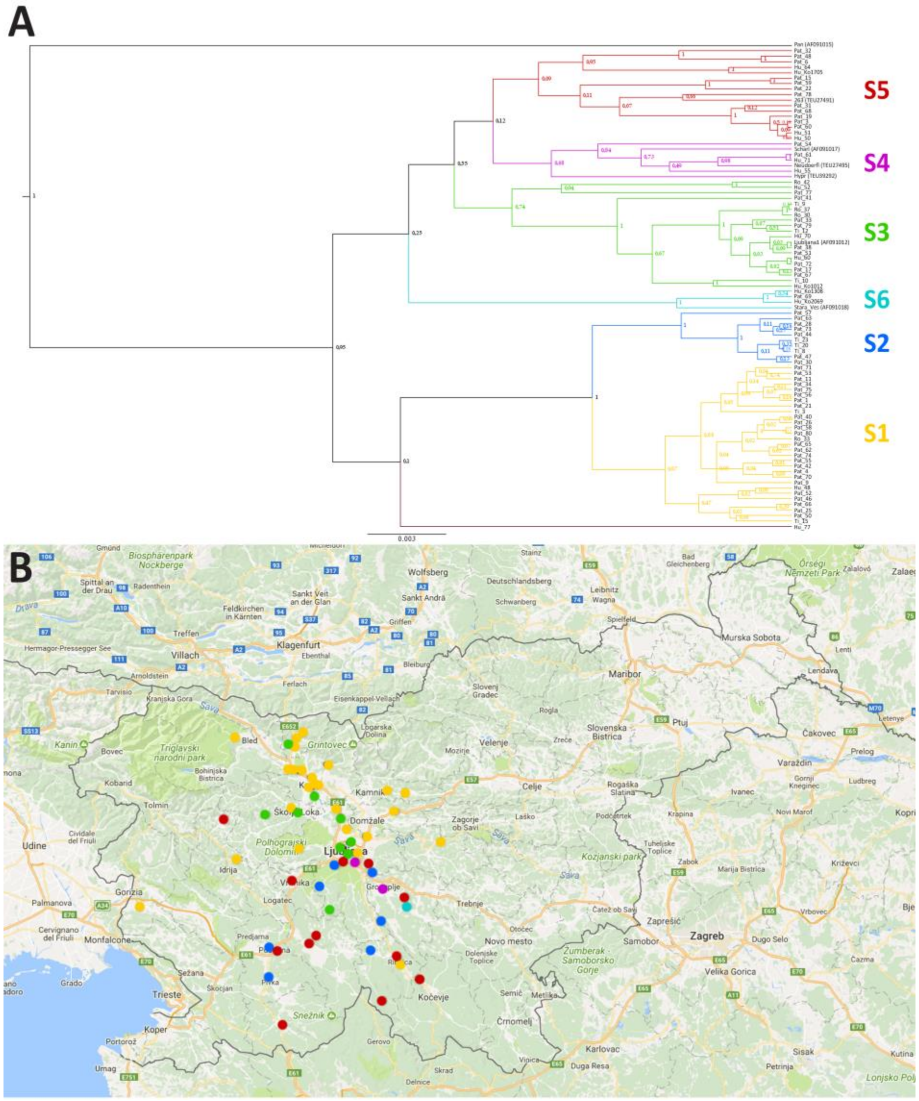

Technical Appendix Figure. Correlation of phylogenetic and geographic clustering of virus RNA sequences of tickborne encephalitis viruses, Slovenia. A) Bayesian phylogenetic analysis of 56 partial envelope protein gene sequences (1,272 bp). Scale bar indicates nucleotide substitutions per site. S, clade. B) Map of Slovenia showing geographic locations (circles) of permanent residencies of patients. Circles are colored according to results of phylogenetic analysis of an envelope protein gene sequence of tickborne encephalitis virus, which identified clades of virus isolated from patients. Blue boxes indicate road numbers.
